# Exploring barriers to social distancing during the COVID-19 pandemic in Zimbabwe: a qualitative study

**DOI:** 10.1136/bmjph-2024-001962

**Published:** 2025-11-05

**Authors:** Masceline Jenipher Mutsaka-Makuvaza, Nicholas Midzi, Lincoln Sunganai Charimari, Priscilla Mangwiro, Gladys Mugadza

**Affiliations:** 1 Department of Microbiology and Parasitology, University of Rwanda, Butare, Rwanda; 2 Ministry of Health and Child Care, Harare, Zimbabwe; 3 World Health Organisation, Harare, Zimbabwe; 4 College of Health Sciences, Harare, Zimbabwe

**Keywords:** COVID-19, Public Health, Zimbabwe, Qualitative Research

## Abstract

**Introduction:**

Social distancing is an effective intervention for reducing the transmission of COVID-19. However, adherence varies across settings. We explored barriers to compliance with Zimbabwe’s social distancing guidelines during the COVID-19 pandemic.

**Methods:**

We conducted a qualitative study with a sample of 30 key informant interviews and 10 focus group discussions with health workers, village health workers, church leaders, traditional healers, teachers, youth leaders, women leaders, transporters and members of general population purposively selected across 10 sites in Zimbabwe during the period of September–October 2022. We audio-taped the sessions, transcribed them verbatim and translated the data into English. We used inductive thematic analysis to code the data and identify themes iteratively.

**Results:**

We generated four themes as barriers to social distancing. These included (1) personal attitudes or beliefs including negative perceptions about COVID-19 preventive measures, low-risk perceptions and lack of knowledge; (2) access-related challenges such as transport challenges, lack of on-premise water, limited household or meeting space, incapacitated quarantine facilities and caregiving a COVID-19 case; (3) the negative impact of social distancing on productivity and livelihoods; and (4) its negative impact on sociocultural and religious beliefs.

**Conclusions:**

Barriers to adherence to social distancing measures, as perceived by Zimbabweans, were personal attitudes or beliefs, socioeconomic, cultural and religious factors. In future pandemics, a tailored approach to public health messaging and interventions is necessary to increase adherence.

**WHAT IS ALREADY KNOWN ON THIS TOPIC**

**WHAT THIS STUDY ADDS**

- This study adds insights into the specific barriers to social distancing in Zimbabwe.

**HOW THIS STUDY MIGHT AFFECT RESEARCH, PRACTICE OR POLICY**

- The study findings improve our understanding of people’s reservations on social distancing measures and might therefore aid with the design of measures that increase acceptability of these measures in future outbreaks.

## Introduction

 The COVID-19 pandemic, declared by the WHO in March 2020, prompted countries worldwide to implement restrictions such as social distancing to reduce viral transmission.^1 2^ Other containment and mitigation measures implemented included isolation, contact tracing and quarantine, hygiene, sanitation, face masking and ventilation.^2^ The success of these measures in reducing transmission depended largely on public compliance, which varied across populations and settings.^3^,^7^ The term *compliance* was used interchangeably with *adherence* in this study to refer to the extent to which individuals follow recommended social distancing during the COVID-19 pandemic. It is acknowledged that the use of these terms has evolved in public health and health psychology; adherence often refers to a positive proactive, voluntary behaviour, whereas compliance is often seen as simply following instructions.^8^ However, our choice of the term *compliance* is consistent with the language used in Zimbabwe’s COVID-19 policy documents.^9^

Globally, research has identified several factors influencing adherence to social distancing, including demographic, psychological and social variables. For instance, personal perceptions, suitable housing infrastructure and trust in government have been linked to compliance.^3^,^6^ A systematic review reported individual-level or community-level psychosocial phenomena and flaws in governmental action or communication as two broad classes of barriers to social distancing across the globe.^7^ In African settings, adherence has been limited by overcrowding, reliance on public transport, social gatherings, informal employment and cultural norms.^10^,^12^

In Zimbabwe, the government imposed a national lockdown on 30 March 2020, with evolving restrictions but maintaining social distancing as a central preventive measure.^13^ In the early stages of the pandemic, public compliance was high,^14^ but it later on reduced due to growing challenges such as economic hardships and limited access to essential supplies.^15 16^ In the city of Harare, overcrowded living houses were cited as one of the factors enabling the spread of COVID-19.^16^ While these studies highlighted essential barriers, they were focused on small communities or were conducted during the early periods of the pandemic.^15^,^20^ This leaves a gap in understanding how social distancing was experienced over time.

This study was conducted nearly 2 years into the pandemic, aiming to explore individual, interpersonal and structural barriers to social distancing among the population in Zimbabwe. Understanding barriers to social distancing in this context is critical for informing the design of socioculturally appropriate and sustainable interventions, upholding public health equity and improving future pandemic response.

To achieve this goal, this study was guided by the Social-Ecological Model (SEM) for Health Promotion, which considers several levels of influence on health behaviour including intrapersonal, interpersonal, institutional, community and public policy.^21^ The model provides context-specific approaches that go beyond individual factors to address social and environmental factors.^10 22^ For example, individual-level factors, such as COVID-19 knowledge and income source, may influence personal risk perception^1022^,^24^; interpersonal influences from family and peers can influence one’s behaviour.^22 23^ Institutional factors, such as lack of supportive environment at learning institutions, workplaces or religious spaces, may hinder adherence.^10 23^ Community norms, values and public policy also affect adherence.^10 22 23 25^ The SEM for Health Promotion framework is useful in the Zimbabwean setting, where socioeconomic limitations, institutional trust, sociocultural beliefs and religious practices differ across communities affecting adherence to public health measures.

## Methodology

### Study design and study population

This study employed a descriptive phenomenological qualitative design, with attention to the social and cultural group contexts in which participants lived. This approach is well suited to exploring individuals’ perceptions and lived experiences within their sociocultural environments, particularly in relation to how they responded to COVID-19 social distancing guidelines. The phenomenological approach^26^ offered the necessary flexibility to capture the dynamic and diverse COVID-19 experiences in urban and rural Zimbabwean settings.

The study was conducted in line with the Standard Consolidated Criteria for Reporting Qualitative Research (online supplemental file 1).^27^ It forms part of a larger multicountry study coordinated by the WHO Regional Office for Africa, which aimed to understand the social and behavioural determinants of population compliance with public health and social measures (PHSMs) and COVID-19 vaccine uptake in six selected African countries.

The study sites and study population have been described previously.^28^,^30^ Briefly, 8 out of Zimbabwe’s 10 provinces were included in the study: Harare, Masvingo, Matabeleland North, Matabeleland South, Midlands, Mashonaland East, Mashonaland West and Mashonaland Central. Study sites within each province were selected based on vaccine uptake statistics from the District Health Information Software 2 (DHIS2) database. Preference was given to districts reporting low vaccine uptake to explore the underlying structural, behavioural, economic and social factors driving low compliance with PHSMs in communities resisting or those at risk of exclusion to public health measures. Different social and economic population groups comprising health workers, village health workers, teachers, traditional healers, transporters, religious leaders, women leaders, youth leaders and the general population aged ≥18 years were selected across the country to capture variation in perceptions and behaviours among the Zimbabwean population. At each study site, participants were grouped homogeneously for both focus group discussion (FGD) and key informant interviews (KIIs).

In Harare Metropolitan Province, three high-density, overpopulated districts—Mbare, Zengeza and Epworth—were selected. In these sites, transporters, religious leaders and women leaders were recruited, respectively. In Mashonaland West Province, Makonde District was selected, and village health workers were recruited in the study. Data collection took place at the district’s growth point, which was formerly a farming compound. In Mashonaland East Province, Seke, a peri-urban district, was chosen as the study site for the general population. In Mashonaland Central Province, Rushinga, a district which borders Mozambique, was chosen for participation in the study. Youth leaders were the participants in this district. Chiredzi district was chosen for participation in the study in Masvingo province. The densely populated town is central to the trade between Harare, Chiredzi, Masvingo towns and South Africa. Traditional healers were the participants in this district. In the Midlands province, Gokwe South district was included, with schoolteachers being recruited for the study. In Matabeleland North Province, Binga District, a resort area attracting tourists and fish traders was selected. In Matabeleland South Province, Insiza District was chosen. Health workers were recruited in Binga, while members of the general population were selected in Insiza.

### Sampling and sample size

At each site, purposive sampling was used to select participants who shared the same characteristics and were likely to provide rich, relevant and diverse data pertinent to the research questions. In each of the 10 selected districts, one FGD was conducted, and three key informants (KIs) were interviewed. Homogeneity among FGD participants was considered to enhance the depth and saturation of data. As described previously,^28^,^30^ we considered participants based on their accessibility, availability and willingness to participate in the study. For each study group, those with first-hand information about their community, fellow community members and COVID-19 issues based on their professional expertise or experience, social positions, participation in a COVID-19 programme in the area and who were non-judgemental and sensitive to differences among community members were recruited as KIs. At each site, 3 KIs were identified giving a total of 30 KIs for the study. Three KIs per site were deemed sufficient to provide in-depth knowledge on community perceptions and experiences. The number per site also guaranteed feasibility of conducting interviews across multiple sites. For the FGDs, individuals who could express themselves comfortably in a group setting to provide diverse COVID-19 perspectives and experiences for the community were selected. One FGD was conducted per each of the 10 sites. While each FGD was expected to have 10 participants, actual group sizes ranged from 9 to 10 participants, which is consistent with qualitative research guidelines recommending 4–12 participants per FGD.^27^ In total, 98 individuals participated in the FGDs.

### Study guides and data collection

The WHO developed study guides based on the literature on compliance with COVID-19 preventive measures. These guides were contextualised for the Zimbabwean settings by the local researchers. Local researchers included a PhD expert in grounded theory and ethnography, PhD holders in community health, individuals with master’s degrees in public health and research assistants with at least a degree in social sciences or public health. The tools consisted of a semistructured KII guide, FGD guide (online supplemental file 2) and observation checklist, which were used to uniformly collect data across all study groups. The FGD and KII guides had similar questions including those seeking to understand general community knowledge, perceptions and experiences with COVID-19 and its preventive measures, and challenges that hindered adherence faced by the community obstructing adherence to COVID-19 preventive measures. Each FGD and KII was conducted in person by two moderators, in English or one of the two local languages, Shona or Ndebele. The FGDs lasted approximately 45 minutes, while KIIs took about 30 minutes.

### Data analysis

Data were analysed thematicallyusing an inductive approach as themes were generated from the data directly without a pre-existing coding framework. All the audio recordings were transcribed verbatim by the moderators at each study site. Recordings conducted in local languages were transcribed verbatim and translated into English. To ensure the accuracy and consistency, the translated scripts were reviewed by an independent language expert. Two authors read and re-read the transcripts, familiarising themselves with the data and generating preliminary codes based on emerging content. The codes were developed from the data reflecting participants’ own words and meanings. To allow close engagement with the data, the coding was conducted manually in Microsoft Word. The dataset was manageable, so it was feasible to code manually. The two initial coders compared and discussed their codes to enhance rigour. Intercoder reliability was ensured through consensus meetings to discuss and resolve coding discrepancies. These initial codes were then reviewed and refined by other authors, who cross-checked them against the transcripts. The research team engaged in iterative discussions of the analysis process, collaboratively reviewing codes, naming and defining themes manually.

### Credibility and trustworthiness

To enhance the credibility of the study, we purposively sampled different population groups with different demographic characteristics including age, socioeconomic status, cultural and religious norms and geographical location. This was intended to reduce selection bias associated with pre-existing social groupings and ensure a diverse range of perspectives on COVID-19 PHSMs. The use of similar questions across the interview and FGD guides enabled triangulation through multiple data sources, strengthening the reliability of the findings. Dependability was enhanced through manual coding which allowed for close interaction and interpretation of participant narratives while also necessitating regular team discussion during the data analysis process. We built rapport and trust with participants to encourage openness and honesty. Participants were ensured of confidentiality throughout the process. To enhance transferability, we have included direct participant quotations and provided a detailed description of the study process from design through analysis.

### Reflexivity and positionality

The local researchers had varying degrees of knowledge of the study communities. Some had prior knowledge of the study communities’ sociocultural and linguistic backgrounds, while others had no prior knowledge of the communities. These positionalities might have influenced interactions with participants and data interpretation.^31 32^ To address this, the researchers maintained a reflexive stance by documenting observations and assumptions during the data collection process. The research team also engaged in discussions to critically reflect on emerging codes and themes, to ensure data interpretations were grounded in participants’ narratives rather than researcher bias.^33^ This iterative and transparent process supported the credibility and integrity of the findings.

###  Ethical approval and consent

Written informed consent was obtained from all participants prior to the start of data collection. Participation was voluntary and respondents were informed of their right to withdraw from the study at any time without penalty. No personal identifiers were included in transcripts or reports, and all data were handled confidentially.

## Results

### Demographics of the study participants

Of the expected sample size of 130 participants, 128 participated in the study. Two individuals, one from traditional healers and the other from women leaders, were unable to attend the FGDs on the scheduled days. The average age of the participants was 45 years (SD=11 years). Among the FGD participants, 50 (51.0%) were males, while 20 (66.7%) of the key informants were males (table 1). Sixty-two (63.3%) of the FGD participants had attained a secondary level of education, and 26 (26.5%) had a tertiary level of education. Among the key informants, 17 (56.7%) had attained a secondary level of education, while the remainder had acquired a tertiary education.

**Table 1:** Gender and level of education among the FGD and KII participants

Variable	District/province	Total	Gender		Level of education		
			Male n (%)	Female n (%)	Primary n (%)	Secondary n (%)	Tertiary n (%)
Overall	All study districts/provinces	128	70 (54.7)	58 (45.3)	10 (7.8)	79 (61.7)	39 (30.5)
KI	All study districts/provinces	30	20 (66.7)	10 (33.3)	0	17 (56.7)	13 (43.3)
FGD participants	All study districts/provinces	98	50 (51.0)	48 (49.0)	10 (10.2)	62 (63.3)	26 (26.5)
**FGD Study group**							
Village health workers	Makonde/Mashonaland West	13	4 (30.8)	9 (69.2)	2 (15.2)	11 (84.6)	0
Health workers	Bimga/Matabeleland North	13	8 (61.5)	5 (38.5)	1 (7.7)	0	12 (92.3)
General population	Insiza/Matabeleland South	13	7 (53.9)	6 (46.2)	4 (30.8)	8 (61.5)	1 (7.7)
General population	Seke/Mashonaland East	13	8 (61.5)	5 (38.5)	0	9 (69.2)	4 (30.8)
Religious leaders	Zengeza/Chitungwiza, Harare	13	10 (76.9)	3 (23.1)	0	6 (46.2)	7 (53.9)
Teachers	Gokwe South/Midlands	13	5 (38.5)	8 (61.5)	0	0	13 (100)
Traditional healers	Chiredzi/Masvingo	12	5 (41.7)	7 (58.3)	1 (8.3)	11 (91.7)	0
Transporters	Mbare/Harare	13	13 (100)	0	0	13 (100)	0
Women leaders	Epworth/Harare	12	0	12 (100)	2 (16.7)	10 (83.3)	0
Youth leaders	Rushinga/Mashonaland Central	13	10 (76.9)	3 (23.1)	0	11 (86.6)	2 (15.4)

Gender distribution adapted from Mutsaka-Makuvaza *et al*.^28^

FGD, focus group discussion; KII, key informant interview.

Four key themes emerged as barriers to social distancing. These were (1) personal attitudes and beliefs including negative perceptions of COVID-19 preventive measures, low risk perception and lack of knowledge; (2) access-related challenges such as transport challenges, absence of on-premise water, limited household or meeting space, inadequate quarantine facilities and the burden of caregiving to a COVID-19 case at home; (3) negative impact on productivity and livelihoods; and (4) negative impact on sociocultural and religious practices.

### Theme 1: personal attitudes or beliefs

#### Negative perceptions about COVID-19 preventive measures

Some resistance to social distancing was brought about by the perception that the measures conflicted with core religious practices such as gathering. Gathering restrictions were not merely taken as public health interventions but a direct threat to collective worship and religious identity. This led to non-adherence particularly among faith-based groups who interpreted the guidelines as undermining religious freedom and communal worship. One participant put it as follows:

… the church initially felt attacked by the new order of life, where people could not gather to fellowship and pray…. COVID-19 prevention measure were seen as eroding believers’ faith and prayer life…. … a phenomenon that had introduced new associations, new beliefs, and new practices antagonistic to the church. FGD participant 2 (religious leader), Zengeza District

#### Lack of knowledge

Social distancing was difficult, particularly in school settings among the young children who had limited understanding of COVID-19 and its preventive measures and naturally tended to engage and play closely. Teachers highlighted that social distancing was feasible in early childhood and primary school settings only under direct supervision. This increased the risk of viral transmission within and between households. One of the participants explained it as follows:

… It worked for adults, but with the little ones it was a big challenge as they always play close to each other and don’t understand the concept. In schools distancing worked in the presence of the teacher only but during break time and lunchtime; it was difficult to enforce. FGD participant 7 (teacher), Gokwe South District

#### Low-risk perception

Despite being educated about the importance of social distancing, adherence in the absence of enforcement was not consistent due to limited internal motivation. Community members would temporarily follow social distance rules when under observation only. Such actions show that there was no lasting behavioural change due to low risk perception and lack of responsibility. One of the participants shared:

… People would just maintain a distance for a short period and get back together. Without someone to enforce, community members could not maintain social distance on their own. FGD participant 3 (community member), Insiza District

### Theme 2: access-related challenges

While negative perceptions about COVID-19 preventive measures, lack of knowledge and low-risk perception about the disease contributed to poor adherence to social distancing, these factors were aggravated by access-related practical barriers including limited transport, infrastructure, overcrowding, poor quarantine facilities and home-based care challenges.

### Limited transport

Participants highlighted that social distancing was a problem because limited transport services created crowding and unsafe conditions. Despite that the government’s transport policies were intended for infection control compliance by some bus operators to reduce capacity in public buses led to long queues and stampedes, undermining social distancing guidelines. Additionally, enforcement of the transport policies forced desperate commuters into informal and riskier modes of transport such as private vehicles, risking both arrest and exposure to COVID-19. A key informant described the situation as follows:

Travelling was a problem. Zimbabwe United Passenger Company (ZUPCO), a government-owned transport was the only means of transport…. Long queues were experienced at the bus terminus. In such situations, observing physical distance became a challenge … and the buses also had limited carrying capacity due to COVID-19 preventive measures. Private transport became our second means of transport, but the police arrested the drivers…. KI 1 (religious leader), Zengeza District

In some areas, some public transport operators did not change their carrying capacity and commuters would find themselves overcrowded in the buses. Non-compliance with social distancing guidelines was systematic. Individuals were pressured to accept unsafe conditions or find unaffordable alternatives. Some of the participants shared the following:

It was bad as there were transport challenges at that time, and we were forced to overload; hence, physical distancing … was a non-event. FGD participant 2 (transporter), Mbare District

In public transport, there were no changes in the carrying capacities, …. You were even told to resort to private transport. FGD participant 2 (women leader), Epworth District

### Congestion at water points and agricultural inputs distribution sites

Communal points like agricultural input distribution sites and boreholes also posed social distancing challenges. Scarcity and urgency made crowd control unmanageable. Thus, essential services points were also high-risk areas prompting the village health workers to focus on harm reduction rather than complete adherence to public health measures. Some of the participants reported as follows:

Social distance was difficult to maintain, especially at boreholes, as people will be scrambling for water. FGD participant 7 (youth leader), Rushinga District

The challenges that we faced in social distancing here in the rural areas were attributed mainly to the crowding of people because of presidential inputs (pfumvudza). Most people do not observe social distancing during these times …. What we would then do is to move around in the crowd, encouraging people to wear their masks properly. KI 1 (village health worker), Makonde District

### Inadequate Infrastructure in public and private environments

Participants noted that public meeting places such as community halls, schools and traditional healing areas, were small and physically unsuited for social distancing. This lack of space made it difficult to separate people even when willing. This suggests that sometimes adherence to social distancing was impractical in real-world settings due to limited physical space. One participant pointed out the following:

The problem with social distancing is that our community halls are small, … the space won’t allow social distancing. The same applies to our schools where the classrooms are small and … cannot accommodate many people. KI 2 (village head), Insiza District

As faith healers, … the house that we lodge will not be able to afford enough space recommended for social distancing…. FGD participant 1 (traditional healer), Chiredzi District

### Overcrowded living conditions

Social distancing was also not practical in urban and informal settlements due to high household density. Multiple families shared single rooms, and isolation within homes was not practical. This highlights that the public health guidelines were not considering real-life overcrowding realities which are often common in low-socioeconomic settings. It was explained as follows by some participants:

… social distance did not work based on the nature of how crowded we are in our residential places. One single room divided by curtains houses four families. FGD participant 7 (community member), Seke District

### Quarantine and home-based care challenges

Social and infrastructural factors also shaped people’s ability to adhere to social distancing guidelines during quarantine. While quarantining was not a challenge for those with large houses or many rooms, it was impractical for families who lived in single rooms. On the other hand, poor logistical planning and coordination at quarantine facilities led to a lack of food and manpower. This undermined public trust and adherence to guidelines. One of the participants shared:

…but for the quarantining, it was not a problem for those with bigger houses; maybe you separate or select one room to be occupied by whoever is affected. But looking at areas where occupants use a single bedroom when they are maybe 3 or 4, it becomes difficult to quarantine somebody …. Apart from that, we had areas that were set up for quarantining those affected, but those areas were not well-manned. … for example, here, … an area like the Cheziya Clinic; you could go there and find no one there or no food provided. …those who wanted to be quarantined would end up running away because no one would survive in the absence of food. KI 1 (schools inspector), Gokwe South District

Household infrastructure was also a significant factor during isolation. The participants noted that the low-income communities found it difficult to adhere to isolation guidelines for COVID-19 since the space for isolation was limited and most people used shared facilities. High-income households with private facilities found it feasible to adhere to guidelines. Thus, disparities in public health measures adherence were not only shaped by willingness but also by economic class. Participants also noted that isolation at institutions was sometimes inconvenient or inaccessible. One of the participants expressed:

Isolation works in some communities. When you get home, say we are isolating you in this room and get to use the same kitchen and the same toilet. That [isolation] works for guys with their en-suites not for us who use the common toilet and for us to say let them be at the hospital; it is also not convenient for some, but maybe we just try to isolate. KI 2 (schools inspector), Gokwe South District

### Theme 3: the negative impact of social distancing on productivity and livelihoods

Beyond the logistical challenges, practising social distancing was further hindered by economic vulnerability of individuals dependent on daily income and the need for continuous learning of students to improve their livelihoods.

### Economic vulnerability

Economic vulnerability also hindered social distancing. Public transport drivers indicated that it was not practical to practise physical distancing due to economic hardships. Despite also being frontline workers, drivers lacked financial compensation which was given to their co-employees. This pressured them to meet targets but compromised compliance to social distancing in the process. This highlights the need for equitable support to all frontline workers during a pandemic to foster better compliance with disease prevention measures. One of the drivers shared:

As drivers we were the hardest hit by the COVID-19 pandemic; we suffered so much because the only transporter ZUPCO, was paying the conductor only and the driver was supposed to be remunerated by your employer. We never got to get the COVID allowance; the conductor only got it. We … started overloading when the police relaxed enforcement since we had targets to meet. There was nothing we could do except work tirelessly since we had no allowances for COVID-19. How was I expected to look after everyone else when the job was not even looking after me? FGD participant 5 (transporter), Mbare District

Maintaining regular customer relations, particularly in the informal sector, was one of the contributing factors to non-adherence to public health measures. Informing customers to practise COVID-19 preventive measures was perceived as rudeness and would result in customer dissatisfaction and consequently low sales. Economic pressure superseded health needs. One participant shared:

Because people want money, their main priority is to make their customers happy. At times you feel that if you tell them to move away, some might think you are rude, affecting customer care. It’s something that we were not used to. That was one of the things that affected us such that you end up ignoring what is required of you. KI 2 (youth leader), Rushinga District

### School-based challenges

Logistical challenges related to social distancing were also experienced in schools, especially those with overcrowded classrooms. The feasibility of implementing social distancing in class was constrained by the educational system’s infrastructural limitations. Social distancing meant that the class size would be reduced and that children would have to attend school on a rotational basis, limiting the time for learning, disrupting syllabus coverage and consequently leading to learning disparities among students.

We had a challenge in class as learners were supposed to sit 1 metre apart. Considering the enrolment here in class where we have large numbers, this meant learners were then coming on a rotational basis; thus, we then had problems covering syllabi, as kids could not fit in class all at once. FGD participant 4 (teacher), Gokwe South District

### Theme 4: the negative impact of social distancing on sociocultural factors and religious beliefs

Other than the negative impact of social distancing on productivity and livelihoods, communities’ failure to adhere to social distance was closely intertwined with its negative impact on sociocultural and religious obligations which often conflicted with public health measures.

### Negative impact on sociocultural practices

COVID-19 preventive measures conflicted with some local sociocultural and religious practices. Some communities stigmatised social distancing. The joy and familiarity among community residents during social gatherings, compounded with deep-rooted customs of social interaction, made behaviour change difficult and slow. Public health interventions were conflicting with social customs. This highlights the significance of culture-sensitive behaviour change policies. One participant explained:

At times, one would forget. When we gather, there is always happiness that people can forget about, ignoring social distancing, greeting each other and so on. That is what l saw. What we do is that we are used to a certain way of doing things, and it is difficult to change; it might take time. KI 2 (youth leader), Rushinga District

Besides cultural habits, social distancing adherence was compromised because those who attempted to adhere would face social backlash in the community. Pressure to conform to social norms superseded pressure to conform to COVID-19 preventive measures. The measures were perceived as offensive in the community, and this forced some individuals to forgo health-positive behaviour due to fear of social rejection or in favour of community identity. One of the participants shared:

Most people didn’t comply with it [social distancing]. It was difficult. Those who tried complying were stigmatised and labelled antisocial. KI 1 (traditional healer), Chiredzi District

Participants expressed that caregiving duties, traditional healing practices and client needs conflicted with social distancing guidelines. Family members are morally expected to provide care to their relatives who are unwell. Traditional healing rituals emphasised collective healing, essentially counterattacking physical distance especially when it’s a family consultation. Thus, social distancing guidelines were not feasible in this context as they did not account for interdependence in communal settings. These realities reflected the need to tailor public health strategies to accommodate practical caregiving difficulties and cultural beliefs.

Adherence to social distancing was difficult, especially among those taking care of the sick, especially when the sick person is insane, very sick or compromised in any way; the carers will not let them be as they would be helpless and hopeless. Hearing impairment among clients violated social distancing as the healer moved closer to the client so that he could be heard. As I said before, the spirit medium does not involve social distancing in healing rituals. In case of consultations, the people would want to receive the message at one point in time, meaning the house will be full and social distance will be compromised. FGD participant 9 (traditional healer), Chiredzi District

Participants mentioned that it was difficult to adhere to social distancing during funerals. Traditional funeral rites such as communal gathering, singing and drumming are central to mourning practices among communities in Zimbabwe. Social distancing guidelines violated these rites which are considered as a sign of collective grieving and mutual support during the death of a community member. One of the participants expressed:

We experienced a lot of challenges, especially during funerals, since in this community, people play drums and sing; so, advocating for social distancing was difficult because people were used to their way of life and controlling people was a big challenge, especially at funerals. KI 3 (village health worker), Makonde District

Another participant also echoed that non-attendance to a funeral in the community was socially unacceptable. The need to show solidarity and avoid being considered as emotionally cold or disrespectful undermined the COVID-19 preventive measures. This suggests the need for approaches which are culturally sensitive in pandemic response policies. One participant shared:

It is not easy to shun a neighbour following a COVID-19-related death even though it is one of the preventive measures. FGD participant 1 (community member), Seke District

### Disruption of informal social and leisure activities

Social distancing was considered as socially disruptive and unwelcome to community informal social gatherings. Places such as beer halls and other leisure settings are fundamental for social closeness, bonding and belonging. In this context, social distancing was viewed as unnatural and conflicted with community norms of engagement and recreation. This suggests the need for careful framing of public health messaging to avoid non-adherence in such contexts. One of the participants expressed the following:

Social distance interfered with socialisation, especially at beer halls and other social circles; adhering to social distance in these circles was not acceptable. FGD participant 8 (youth leader), Rushinga District

### Negative impact on religious spaces and emotional reunions

Participants highlighted that there were space limitations in churches which made social distancing impractical. Practising social distancing in church would go against the spirit of collective fellowshipping. In addition, during family reunions, the emotional need for closeness and social expectations when welcoming relatives overshadowed perceived risk of COVID-19 transmission, thus weakening people’s adherence to social distancing. One participant explained:

Yeah, it was not easy to maintain social distance, especially in the Church, like where we are now with these few little benches (participant pointing to the small benches), where the rest of the congregants sit if we maintain social distance? Additionally, social distancing was a challenge, especially when individuals returned from abroad, and all the family members wanted to sit close by them and sometimes they had not been screened for the COVID-19 virus. People would hug, …without thinking of the possibility of the COVID-19 virus spreading. FGD respondent 2 (village health worker), Makonde District

Taken together, these four themes confirm that compliance to social distancing was shaped by a multifaceted interaction of individual factors, structural and economic limitations, sociocultural and religious norms. The findings highlight that social distancing was not only a personal choice but rather a complex behavioural challenge influenced by cultural values and socioeconomic realities.

## Discussion

Among the subpopulations studied in Zimbabwe including health workers, village health workers, church leaders, traditional healers, teachers, youth leaders, women leaders, transporters and members of general population, non-adherence to social distancing was driven by access-related challenges, sociocultural and religious beliefs, negative effects of social distancing on productivity and livelihood, negative perceptions about COVID-19 preventive measures, lack of knowledge and low-risk perceptions. Among these barriers, access-related challenges and economic constraints emerged as the most significant barrier, and they were frequently mentioned by participants, emphasising its practical implications on daily adherence. Sociocultural and religious beliefs were also a highly influential barrier to social distancing as the preventive measure affected communities’ deeply ingrained practices. While personal attitudes and beliefs and the negative effects of social distancing on productivity and livelihood were also important barriers to adherence, they appeared to be ancillary to physical and livelihood challenges.

### Personal attitudes and beliefs

The study findings showed that congregants perceived that social distancing practices were antagonistic to religious practices. This perception was worsened when lockdown measures were relaxed to reopen social venues such as bars, while church gatherings remained closed, and this created feelings of unfairness. In such scenarios where public health measures are being undermined by incorrect beliefs, it is important to engage religious and community leaders to address wrong perceptions, misinformation and nurture accountability.^34^

Despite being taught COVID-19 preventive measures, young children were reported to have adherence challenges. It was noted that they could not fully understand the measures and would require constant supervision to comply. Public health messages that are understandable across different population groups are therefore critical to uphold adherence. Caregivers and teachers can also adopt strategies to emphasise adherence among the young children. Likewise, due to low-risk perception, some adults would fail to adhere to social distancing even after receiving health education about the disease. These findings support the SEM’s focus on the individual level, where personal knowledge, beliefs and risk perception also contribute to health behaviours.^21^ The findings also align with systematic reviews showing that knowledge, misconceptions and perceived risk considerably influence compliance to COVID-19 preventive behaviours.^7 35 36^ Low-level adherence due to lack of knowledge was also noted in other low-income settings such as Ghana and Ethiopia.^10^ ^11^ Similar to the Zimbabwean setting, in South Africa it was noted that despite the high knowledge of COVID-19 and its preventive measures, low-risk perception was also evident among the population.^37^ This affirms that behaviour change cannot be achieved simply through one-way information dissemination approach via radios and posters but requires two-way engagement through community dialogues with public health promoters addressing affective factors^38^ to improve adherence to preventive measures.

Despite the fact that personal attitudes and beliefs influenced compliance, their impact was less prominent compared with structural and economic challenges, as even individuals with knowledge found it difficult to comply because of these practical barriers.

### Access-related challenges

Access-related challenges proved to be the most dominant barrier as inadequate space in household and public infrastructure, and limited water supplies resulted in unintentional non-compliance to social distancing. It was difficult to adhere to social distancing while using public transport, as operators disregarded regulations by overloading to maximise profits. Families also bemoaned the lack of social distancing due to overcrowding in households or residential areas, resulting in a lack of quarantine and isolation facilities if the household had contact with or had contracted COVID-19. Traditional healers also expressed that their housing facilities were very small for physical distancing, especially in cases where they had to attend to a high number of clients at the same time. These findings corroborate systematic reviews showing that limited infrastructure and overcrowding limited the feasibility of social distancing during the pandemic.^36 39^ Insufficient water, sanitation and hygiene facilities in low- and middle-income settings caused overcrowding and long waiting times at shared facilities, undermining social distancing guidelines.^39^ Comparable studies in poor communities in Ghana and South Africa suggest that in some circumstances, despite people’s willingness to engage in behavioural change, a lack of infrastructure and poverty led to non-adherence with governmental COVID-19 regulations.^40^ A study in Ghana also corroborated our findings, noting that the lack of social amenities contributed to the poor observance of COVID-19 prevention measures.^25^ Similarly, others’ research has also reported non-feasibility of preventive measures based on the environment in ethnic minorities.^41 42^ Unlike in Ethiopia, where low adherence was mainly shaped by age, lack of knowledge and other demographic factors,^11^ our study findings show that in addition to these barriers, structural barrier-related limited infrastructure, access to daily water and economic challenges mainly made behaviour change impractical. A study in the UK reported that individuals were able to comply with social distancing due to the availability of a spacious environment.^4^ In the presence of these infrastructural shortages in Zimbabwe, adherence to public health measures by low-income communities is largely unattainable unless these barriers are addressed. This reflects the influence of interpersonal level of the SEM, where an individual or community may be limited to adhere due to their environmental conditions regardless of intent.^22 39^

### Negative impact on productivity and livelihoods

The economic impact of social distancing was often cited as one of the major drivers of non-compliance, especially among the small business owners and informal workers. Business owners could not adhere to social distancing due to fear of losing customers. Meanwhile, public transport drivers often overloaded vehicles and disregarded social distancing to increase their income. Similarly, challenges in food availability at government quarantine facilities created hardships leading to non-adherence to social distancing. Studies during the Ebola outbreak also noted the need for the provision of necessities such as food to increase adherence to preventive measures.^34 43^ In corroboration with our findings, a systematic review showed that economic vulnerability compromises COVID-19 preventive guidelines.^36^ Despite the fact that economic vulnerability has been noted as a significant barrier in many settings, the heavy reliance of the Zimbabwean population on informal trade magnifies this challenge compared with other settings. The pressure of sustaining livelihoods was high compared with that of adhering to COVID-19 preventive measures, underscoring the priority given to survival needs over public health issues. In the absence of financial cushioning, social distancing became impractical. To ensure that the intervention burden of preventive measures does not outweigh the benefits, a thorough understanding of how the measures work in different contexts, combinations and duration is key. Adherence can be motivated through offering incentives for financial stability and community resourcing.^44^ This situates the theme within the public policy level of SEM and highlights the implications of policy decisions on individual adherence. Meanwhile, the limitations on number school infrastructure reflect the way institutional environment at learning institutions shaped students’ adherence to social distancing guidelines.

### Negative impact on sociocultural and religious practices

While access-related challenges and economic vulnerability were the key practical barriers, the communities’ sociocultural norms and values posed a tough social pressure against adherence, mainly during funeral or religious gatherings. The introduction of social distancing, as a measure for preventing COVID-19 transmission, did not align well with some Zimbabwean cultural norms and values. Physical closeness at funerals, church gatherings and other meetings is an expression of solidarity. Attending funerals and other community gatherings is a cultural norm in Zimbabwe. During such gatherings, people are involved in handshaking, hugging, singing and dancing, and failure to participate is usually met with social ostracism by the community. This makes non-compliance with COVID-19 protocols socially acceptable. Affirming our results, systematic reviews also emphasised sociocultural norms, religious practices and the dependence on social networks as barriers to social distancing.^7 36^ The pattern is also consistent with other African and Asian contexts showing that communities value their norms and may sometimes prefer non-adherence in favour of their cultural practices.^23 25 43 45 46^ Applying the SEM, these sociocultural barriers function at community level, highlighting the need for context-specific messaging and interventions to improve compliance. On the other hand, religious practices reflect institutional barriers, emphasising the significance of engaging faith-based leaders in designing religion-acceptable interventions. Without culture and religious-embedded interventions, sociocultural norms will always be an obstacle to public health interventions.

### Integration of themes with SEM for health promotion

Our study was guided by the SEM for health promotion which theorises that individual health behaviours are designed by multiple networking levels of influence. Barriers to social distancing were noted across the different levels of the SEM framework. At the individual level, despite the government-sponsored awareness campaigns that were consistently conducted during the pandemic, adherence was compromised by limited knowledge, negative attitudes and low-risk perception. At the interpersonal level, social distancing guidelines were undermined by caregiving responsibilities, shared water facilities and inadequate infrastructure and public transport. Meanwhile, at the community level, sociocultural norms pressured individuals to forgo social distancing guidelines in preference for community rituals and gatherings. At institutional level, students could not practise social distancing due to inadequate learning space while religious practices of fellowshipping together undermined social distancing in churches. At national level, the policy which allowed differential remuneration of public transport workers and lack of financial support to informal business and workers resulted in economic vulnerability and consequently undermined social distancing guidelines.

These multilevel insights show that one level interventions such as awareness campaigns will not bring about sustained behavioural change. This underscores the need for interventions across all levels, through improving public health messaging, engagement of communities and their leadership to design feasible, acceptable interventions and providing livelihood support to vulnerable communities during public health emergencies. Context-specific multilevel interventions will increase community acceptance and resilience during public health emergencies.

### Policy recommendations

Our study findings emphasise the need to implement multilevel public health measures to improve adherence to social distancing guidelines during pandemics. At the individual level, awareness campaigns should adopt a two-way communication system to improve knowledge, address misconceptions and low perceptions of risk while building trust in public health interventions. At an interpersonal level, the government should invest in providing sufficient public infrastructure including transport and spacious venues for meetings, adequate water sanitation and hygiene facilities and improve access to health services including quarantine and isolation facilities. At community level, there is a need to engage community leaders during the design of interventions so that the preventive measures are aligned with community norms and values. At institutional level, institutional leaders should also be engaged so that they become accountable and interventions at institutional level are practical. To reduce the economic burden of adherence to preventive measures, the government should come up with a social protection scheme to cushion vulnerable populations and informal workers during public health emergencies.

### Study limitations

Our study had some limitations. First, it was conducted in 10 sites across the country; thus, our findings may be context specific and limited in generalisability. Second, while purposive selection of participants is appropriate in qualitative studies, it may have introduced selection bias as individuals willing to participate may have different views from those that declined to participate. Additionally, social desirability bias may influence reliance on self-reported data. Despite the fact that data collection was performed by trained personnel and supervised to ensure consistency, variations in interpretation of interview questions and interviewer dynamics cannot be ruled out. Finally, COVID-19 is continuously evolving and community perceptions may have evolved since the data were collected.

## Conclusion

The study findings show that awareness alone is not sufficient for adherence to social distancing. Factors such as socioeconomic vulnerability and sociocultural norms may undermine public health interventions. This highlights the need for a tailored multilevel approach to public health messaging and interventions to improve population adherence and resilience during future public health emergencies.

## Supplementary material

10.1136/bmjph-2024-001962 online supplemental file 1

10.1136/bmjph-2024-001962 online supplemental file 2

## Data Availability

Data are available upon reasonable request.
