# Supplementary material for: Exploring barriers to social distancing during the COVID-19 pandemic in Zimbabwe: a qualitative study
Source: BMJ Public Health. 2025 Nov 5;3(2):e001962. doi: 10.1136/bmjph-2024-001962 (PMC12593499; doi:10.1136/bmjph-2024-001962)
Supplement: online supplemental file 1 [file bmjph-3-2-s001.docx]

# Supplementary File 1 - Standards for Reporting Qualitative Research (SRQR) Checklist

| **Index** | **Topic** | **Page(s)** |
| --- | --- | --- |
|  | **Title and Abstract** |  |
| 1 | Title | 1 |
| 2 | Abstract | 1-2 |
|  | **Introduction** |  |
| 3 | Problem formulation | 3-4 |
| 4 | Purpose or research question | 4 |
|  | **Methods** |  |
| 5 | Qualitative approach and research paradigm | 5 |
| 6 | Researcher characteristics and reflexivity | 7,9 |
| 7 | Context | 5-6 |
| 8 | Sampling strategy | 6-7 |
| 9 | Ethical issues pertaining to human subjects | 9 |
| 10 | Data collection methods | 7 |
| 11 | Data collection instruments and technologies | 7 |
| 12 | Units of study | 7, 10, 11 |
| 13 | Data processing | 8 |
| 14 | Data analysis | 8 |
| 15 | Techniques to enhance trustworthiness | 8-9 |
|  | **Results** |  |
| 16 | Synthesis and interpretation | 12-22 |
| 17 | Links to empirical data | 12-22 |
|  | **Discussion** |  |
| 18 | Integration with prior work, implications, transferability, and contribution(s) to the field. | 23-29 |
| 19 | Limitations | 29 |
|  | **Other** |  |
| 20 | Conflicts of interest | 30 |
| 21 | Funding | 30 |

**Reference:** O’Brien BC, Harris IB, Beckman TJ, et al. Standards for reporting qualitative research: A synthesis of recommendations. *Acad Med.* 2014;89:1245–51.
