# Supplementary material for: Exploring barriers to social distancing during the COVID-19 pandemic in Zimbabwe: a qualitative study
Source: BMJ Public Health. 2025 Nov 5;3(2):e001962. doi: 10.1136/bmjph-2024-001962 (PMC12593499; doi:10.1136/bmjph-2024-001962)
Supplement: online supplemental file 2 [file bmjph-3-2-s002.docx]

**KEY INFORMANT INTERVIEW GUIDE**

**Project Title: QUALITATIVE STUDY ON SOCIAL-BEHAVIOURAL DETERMINANTS OF POPULATION COMPLIANCE WITH PUBLIC HEALTH AND SOCIAL MEASURES (PHSM) AND COVID-19 VACCINE UPTAKE.**

1. **Identification**
   1. Age ___________________
   2. Sex ___________________
   3. District ___________________

1.4. Province ___________________

1.5. Country ___________________

1.6. Occupation/Social Position ___________________

1.7. Educational level ___________________

1. **COVID-19 in general**
   1. What do you think COVID-19 is?
   2. Do you believe that COVID-19 exists?
   3. According to your own experiences, what do those around you think COVID-19 is?
   4. In your opinion, which communication channels are best suited for COVID-19 information in this community. Please justify your responses

- Call centres [ ]
- Radio/community radio [ ]
- Television spots [ ]
- Social media [ ]
- Community dialogues and meetings [ ]
- Print media (posters, pamphlets) [ ]
- Others (Please specify) ------------ [ ]

1. **Compliance to COVID-19 PHSM**
   1. What recommended preventive measures (public health and social measures – PHSM), against COVID-19 do you know?
   2. What are your views concerning the usefulness of these PHSM in fighting COVID-19?
   3. According to your experiences, what challenges do people face in undertaking the following recommended preventive measures? *(Please clarify)*
2. Proper use of facemasks
3. Physical distancing
4. Hand hygiene
   1. According to your own experiences, are there any categories of people in this community that you think are less likely to comply with the recommended COVID-19 preventive measures (PHSM)?

- If Yes, please specify
  1. Have you seen any challenges that have been brought about by compliance to any of the recommended preventive measures for COVID-19 in this community? If any, please specify the challenge(s)

1. **Compliance to COVID-19 vaccines**
   1. In your own opinion, what are the benefits of COVID-19 vaccines against COVID-19?
   2. According to your understanding, should COVID-19 vaccines be used against COVID-19?

- If not, please explain your reason(s)?
  1. From your own experiences in this community, what are the reasons why people would refuse to be vaccinated against COVID-19?
  2. Are there any challenges that people have faced in trying to get vaccinated? Please explain

1. **Key influencers in promoting uptake of PHSM and COVID-19 vaccines**
   1. Are there any persons or groups of people in this community, who have been influential in promoting uptake of preventive measures against COVID-19?

- If Yes, please name these (social role/occupation) by their key roles in promoting preventive measures
  1. Are there any persons or groups of people in this community, who have been influential in promoting uptake of COVID-19 vaccines?
- If Yes, please name these (social role/occupation) by their key roles in promoting vaccine uptake in this community.

1. **Recommendations to improve compliance to PHSM and uptake of vaccines**
   1. According to your own experiences, what measures do you think will work in preventing COVID-19 transmission in this community?
   2. In your opinion, what do you think should to be done to make people comply with recommended COVID-19 preventive measures (PHSM)?
   3. In your opinion, who should promote compliance with COVID-19 preventive measures (PHSM) and uptake of vaccines?
   4. According to your own experiences, what measures do you think will work in managing people with COVID-19 in this community?
   5. What do you think needs to be done to get people accept to be vaccinated against COVID-19?

**FOCUS GROUP INTERVIEW GUIDE**

**Project Title: QUALITATIVE STUDY ON SOCIAL-BEHAVIOURAL DETERMINANTS OF POPULATION COMPLIANCE WITH PUBLIC HEALTH AND SOCIAL MEASURES (PHSM) AND COVID-19 VACCINE UPTAKE.**

1. **Identification of participants**
   1. Age _________________________
   2. Sex _________________________
   3. District _________________________
   4. Province _________________________
   5. Country _________________________
   6. Occupation/social role _________________________
   7. Educational level _________________________
2. **Compliance to COVID-19 PHSM**
   1. In your own understanding, what do you think COVID-19 is?
   2. According to what you have heard, what do those around you think COVID-19 is
   3. What recommended preventive measures (public health and social measures – PHSM), against COVID-19 do you know?
   4. In your opinion, what is the importance of these PHSM in fighting COVID-19?
   5. According to your experiences, what challenges have you faced in undertaking the following recommended preventive measures?
3. Proper use of facemasks
4. Physical distancing
5. Hand hygiene
6. **Compliance with COVID-19 vaccines**
   1. In your own opinion, what are the benefits of COVID-19 vaccines?
   2. According to your understanding, should COVID-19 vaccines be used against COVID-19?

- If not, please explain your reason(s)?
  1. From your own experiences in this community, what are the reasons why people would refuse to be vaccinated against COVID-19?

1. **Key influencers in promoting uptake of PHSM and COVID-19 vaccines**
   1. Are there any persons or groups of people in this community, who have been influential in promoting uptake of preventive measures against COVID-19?

- If Yes, please name these (social role/occupation) by their key roles in promoting preventive measures
  1. Are there any persons or groups of people in this community, who have been influential in promoting uptake of COVID-19 vaccines?
- If Yes, please name these (social role/occupation) by their key roles in promoting vaccine uptake in this community.

1. **Recommendations to improve compliance to PHSM and vaccines**
   1. According to your own experiences, what measures do you think will work in preventing COVID-19 transmission in this community?
   2. In your opinion, what should to be done to make people comply with recommended COVID-19 preventive measures (PHSM)?
   3. In your opinion, who should promote compliance with COVID-19 preventive measures (PHSM)?
   4. According to your own experiences, what measures do you think will work in managing people with COVID-19 in this community?
   5. What do you think needs to be done to get people accept to be vaccinated against COVID-19?
